# Selenium-binding protein 1 in head and neck cancer is low-expression and associates with the prognosis of nasopharyngeal carcinoma

**DOI:** 10.1097/MD.0000000000004592

**Published:** 2016-09-02

**Authors:** Fasheng Chen, Chen Chen, Yangang Qu, Hua Xiang, Qingxiu Ai, Fei Yang, Xueping Tan, Yi Zhou, Guang Jiang, Zixiong Zhang

**Affiliations:** a Department of Otolaryngology Head and Neck Surgery, Central Hospital of Enshi Autonomous Prefecture, Enshi Autonomous Prefecture, Hubei Province; b Research institute of Otolaryngology Head and Neck Surgery, Renmin Hospital of Wuhan University, Wuhan; c Affiliated Hospital of Xuzhou Medical College, Xuzhou, Jiangsu Province, China.

**Keywords:** GEO dataset, HNSCC, IHC, overall survival, SELENBP1

## Abstract

**Background:**

Selenium-binding protein 1 (SELENBP1) expression is reduced markedly in many types of cancers and low SELENBP1 expression levels are associated with poor patient prognosis.

**Methods:**

SELENBP1 gene expression in head and neck squamous cell carcinoma (HNSCC) was analyzed with GEO dataset and characteristics of SELENBP1 expression in paraffin embedded tissue were summarized. Expression of SELENBP1 in nasopharyngeal carcinoma (NPC), laryngeal cancer, oral cancer, tonsil cancer, hypopharyngeal cancer and normal tissues were detected using immunohistochemistry, at last, 99 NPC patients were followed up more than 5 years and were analyzed the prognostic significance of SELENBP1.

**Results:**

Analysis of GEO dataset concluded that SELENBP1 gene expression in HNSCC was lower than that in normal tissue (*P* < 0.01), but there was no significant difference of SELENBP1 gene expression in different T-stage and N-stage (*P* > 0.05). Analysis of pathological section concluded that SELENBP1 in the majority of HNSCC is low expression and in cancer nests is lower expression than surrounding normal tissue, even associated with the malignant degree of tumor. Further study indicated the low SELENBP1 expression group of patients with NPC accompanied by poor overall survival and has significantly different comparing with the high expression group.

**Conclusion:**

SELENBP1 expression was down-regulated in HNSCC, but has no associated with T-stage and N-stage of tumor. Low expression of SELENBP1 in patients with NPC has poor over survival, so SELENBP1 could be a novel biomarker for predicting prognosis.

## 1 Introduction

Selenium (Se) is an essential and unique micronutrient for a number of biological processes; deficiency of dietary selenium is associated with an increased incidence of cancers in epidemiological and clinical trial.^[1]^ Selenium exerts its anticarcinogenic effects mainly through selenoproteins at nutritional levels; 25 selenoproteins have been identified in the human genome at least.^[2,3]^ Generally, selenoproteins can be classified into 3 categories.^[4]^ First, there are selenoproteins that have incorporated Sec under a precise process requiring the UGA codon. Second, there are proteins that contain selenomethionine (SeMet) and Sec substitute for cysteine and methionine randomly. Finally, there are selenium-binding proteins (SELENBP), which have been shown to bind selenium covalently.^[5]^ Taken together, selenoproteins are widely involved in the metabolism of cells, and may be related with occurrence and development of cancer.

Selenium-binding protein 1 (SELENBP1, SBP1, hsP56), a member of selenoproteins family, has been shown to mediate the intracellular transport of selenium.^[6,7]^ The gene is located at chromosome 1q21–22; the mRNA is composed of 1721 nucleotides and encoding 640 amino acids. It is expressed in a variety of normal tissue types (liver, heart, lung, kidney) and located in the nucleus and/or cytoplasm depending on cell type, degree of differentiation, and environmental signaling,^[8]^ but its expression is reduced markedly in many types of cancers compared with their corresponding normal tissues, including those cancer of the prostate,^[9]^ gastric,^[10]^ ovarian,^[11]^ lung,^[12]^ colon,^[13]^ and thyroid.^[14]^ Additionally, data recently have indicated that SELENBP1's downregulation may play a critical role in the occurrence and prognosis of cancer; low SELENBP1 expression levels are associated with poor patient prognosis, and further study found that SELENBP1 could be associated with tumor cell proliferation, invasion, metastasis, and resistance to treatment.^[15]^

To get a more comprehensive understanding of SELENBP1 functions on cancer, many researchers have accomplished some in-depth studies. SELENBP1 might exert its tumor-suppressive function as a regulator of the tumor redox microenvironment because decreased expression of SBP1 could affect both GPX1 activity and HIF-1a expression.^[16]^ von Hippel-Lindau protein (pVHL)-interacting deubiquitinating enzyme 1 (VDU1) was recently found as another protein partner of SELENBP1, which was involved in ubiquitination or deubiquitination and mediated protein degradation pathways.^[17]^ Some researchers have noted that SBP1 expression could cause ≥1 of the lipid/glucose metabolism-related proteins changes, such as Dickkopf-related protein 1 (DKK1), Annexin A4 (ANXA4), or nuclear factor Kb (NF-kB), and any one of these molecules plays critical role in carcinogenesis and progression through regulating downstream targets.^[18]^ However, SELENBP1 expression profiling as one of the prediction for patients with cancer remains uncertain.

Head and neck squamous cell carcinoma (HNSCC) is among the 10 most common forms of cancer,^[18]^ including laryngeal cancer (LC), oral cancer (OC), tonsil cancer (TC), hypopharyngeal cancer (HPC), nasopharyngeal carcinoma (NPC), and so on, which have different pathological types, malignant degree, and prognosis. Nasopharyngeal carcinoma (NPC) is endemic to Southeast Asia and the southern part of China.^[19]^ The American Joint Committee on Cancer (AJCC) TNM classification system is widely applied to NPC and predicted the clinical outcome, but accurately predicting the prognosis of NPC is difficult.^[20]^ The histopathological characteristics play an important role in prediction of patient outcomes; SELENBP1 have been noted that is associated with poor prognosis of others cancer patients in the literature, so we try to explore the expression of SELENBP1 in HNSCC and further study of the expression of SELENBP1 correlated with the prognosis of NPC, incorporation of these additional prognostic factors may be helpful to improve its prediction accuracy.

## 2 Methods

### 2.1 Tissues, chemicals, and antibodies

All pathological specimens of HNSCC were diagnosed by pathological examination of a hematoxylin and eosin-stained frozen tissue sections and stored by paraffin embedding in Enshi prefecture central hospital, including 99 cases of NPC, 24 cases of LC, 22 cases of OC, 16 cases of TC, 34 cases of HPC, and 40 cases of control tissues (Table 1). Among them, 99 cases of NPC have detailed follow-up, the others are randomly. An independent set of paraffin-embedded archival tissue specimens used for SELENBP1 immunohistochemical staining using a standard immunohistochemical technique.

**Table 1:**
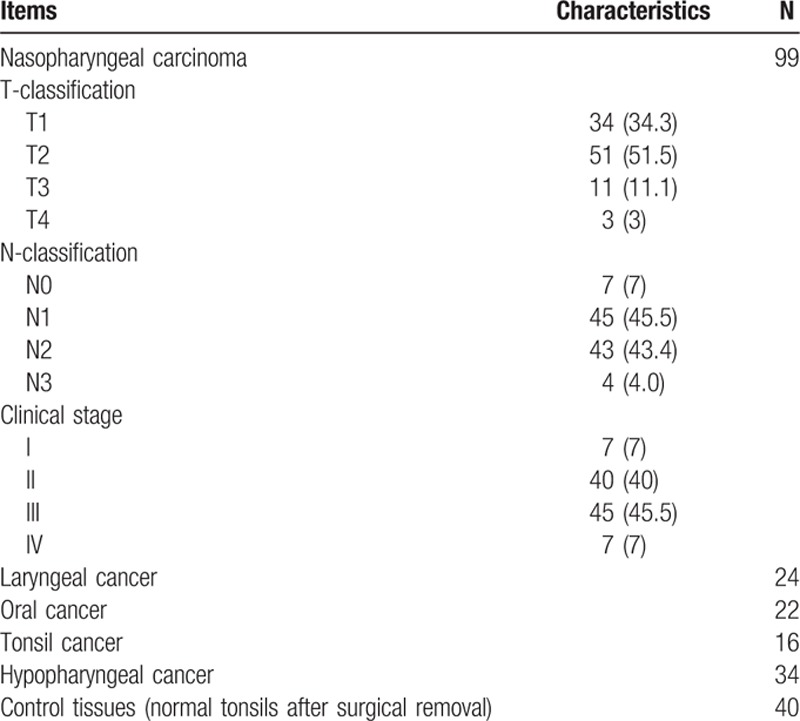
Characteristics of patients selected.

Immunohistochemistry (IHC) was performed to all paraffin-embedded tissue sections; IHC image of the staining was performed on a Leica Bond^TM^ system using the standard protocol. The section was pretreated using heat-mediated antigen retrieval with sodium citrate buffer (pH6, epitope retrieval solution 1) for 20 minutes, then incubated with primary anti-SELENBP1 antibody (ab90135, Abcam) 5 μg/mL for 15 minutes at room temperature and after 3 washes with PBS; each slide was incubated for 3 minutes in 2% 3,30-di-aminobenzidine tetrahydrochloride and 50 mmol/L tris-buffer (pH7.6) containing 0.3% hydrogen peroxidase as a chromogen.

#### 2.1.1 IHC scoring

The staining was blindly evaluated by 2 independent pathologists without knowledge of patient characteristics and a consensus was provided on staining patterns by light microscopy. A quantitative score (- ∼ + +) was performed by adding the score of staining area and the score of staining intensity for each case to assess the expression levels of SELENBP1 protein as previously described by us.^[21]^ A combined staining score – and ± was considered to be low-staining (low-expression); a score between + and ++ was considered to be strong-staining (high expression).

### 2.2 Patients and samples

#### 2.2.1 Ethics statement

The Ethical Committee of Enshi prefecture central hospital approved the study protocol, and the study was conducted in accordance with the principles of the Declaration of Helsinki regarding research involving human subjects. All patients provided written informed consent to participate in this study.

#### 2.2.2 Inclusion and exclusion criteria

The inclusion criteria were as follows: patients younger than 70 years; patients who had undergone a nasopharyngeal biopsy for the diagnosis of undifferentiated NPC; patients without distant metastasis; patients who had undergone conventional chemoradiotherapy; patients with adhering to follow-up; patients died because of nasopharyngeal carcinoma; patients who understood and voluntarily signed an informed consent form.

Patients with NPC (n = 99) who underwent conventional chemoradiotherapy at Enshi Prefecture central hospital (Hubei Province, China) were enrolled in this study. Tumor paraffin-embedded specimens used in IHC analyses were consecutively chosen from 99 patients between 2006 and 2011. All Patients were followed up every 3 to 6 months after chemoradiotherapy until June 15, 2015. The median follow-up period was 63 months (range, 16–126 months). Overall survival (OS) was defined as the interval between end of the treatment and death or between end of the treatment and the last observation point.

### 2.3 Data preprocessing

The microarray data of GSE33205, GSE6631, GSE59102, and GSE39366 were downloaded from Gene Expression Omnibus (GEO) database (Table 2), the largest comprehensive public functional genomics data repository. A total of 138 cancer samples, including 29 Larynx squamous cell carcinoma, 30 margin samples, and 22 normal samples were collected. By taking the average expression value, the expression values of all probes for each gene were converted to a single value. After the gene expression was analyzing by Affymetrix Genechip System (Affymetrix, Santa Clara, CA), with *t* test and variance analysis being identified, *P* < 0.05 was considered to the differentially expressed genes.

**Table 2:**
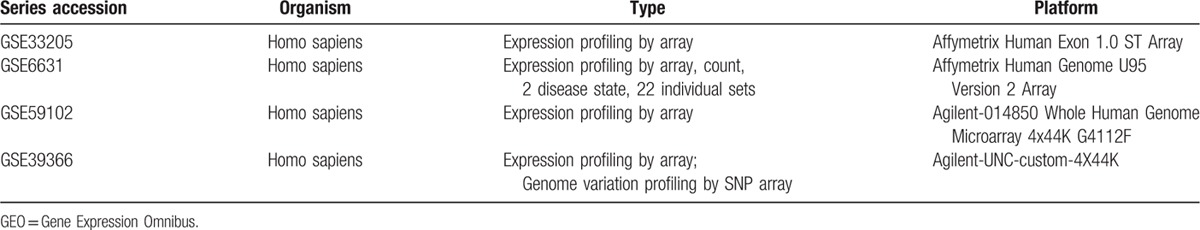
Details of information of GEO dataset.

### 2.4 Statistical analysis

The differences of SELENBP1 expression between HNSCC and normal tissues were assessed using a 2-sample *t* test. The Kaplan–Meier method was used to assess differences in patient metastasis–free survival, recurrence–free survival, and OS. Binary logistic regression analysis was used to assess the relevance of SELENBP1 expression and tumor N-stage, T-stage, and tumor grade. Statistical analyses were performed using SPSS 21.0 software (IBM Corporation, Armonk, NY). *P* value <0.05 was considered statistically significant.

## 3 Results

### 3.1 Analysis of GEO dataset

**Figure 1:**
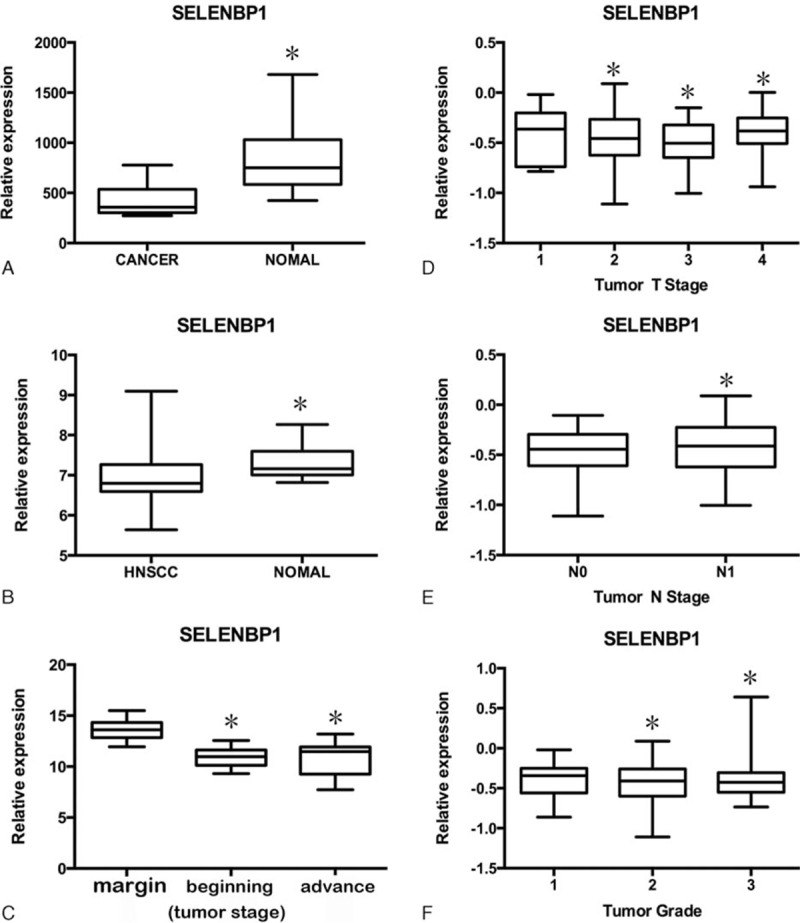
Analysis of SELENBP1 gene expression in cancer and normal tissue from Gene Expression Omnibus (GEO) dataset. (A) Expression of *SELENBP1* gene is relatively low in total cancer in comparison with normal tissue. (B) Expression of *SELENBP1* gene is relatively low in head and neck squamous cell carcinoma (HNSCC) compared with normal tissue. (C) Expression of *SELENBP1* gene in beginning and advanced tumor stage is lower than margin of tumor. (D) Expression of *SELENBP1* gene in different T-stage of HNSCC has no statistically significance. (E) Expression of *SELENBP1* gene in different N-stage of HNSCC has no statistically significance. (F) Expressing of *SELENBP1* gene in different grade of HNSCC has no statistically significance.

GEO dataset GSE6631 included 44 paired (from the same patient) samples of HNSCC and normal tissues were studied with Affymetrix U95A chips. The relative expression of SELENBP1 gene in the 2 groups showed that it was lower in tumor than in normal tissue (*P* < 0.01) (Fig. 1A). Then, data of paired samples from GEO dataset GSE33205 were analyzed, showing the same outcome (Fig. 1B). Twenty-nine cancer samples and 30 margin samples were collected from patients undergoing surgical ablation of Larynx squamous cell carcinoma, the relative expression of SELENBP1 gene was lower in beginning and advance of tumor stage than in margin (*P* < 0.01) (Fig. 1C). These data indicated that *SELENBP1* gene was downregulated in HNSCC.

We further investigated the SELENBP1 expression in different T-stage, N-stage and grade in patients with HNSCC. Data from GEO dataset GSE39366 showed that there was no statistically significant difference (*P* > 0.05) (Fig. 1D, Fig. 1E, and Fig. 1F). These results showed that SELENBP1 expression in HNSCC tissues has no correlation with tumor T-stage, N-stage, and tumor grade. All in together, GEO dataset indicated that SELENBP1 expression in HNSCC was downregulated significantly, but there are no new changes with tumor development.

### 3.2 SELENBP1 expression in the majority of HNSCC is low expression

**Figure 2:**
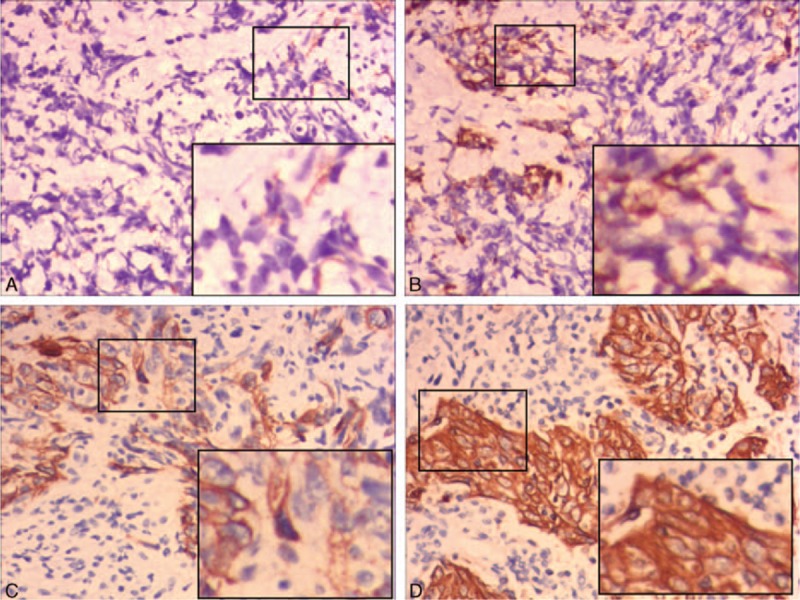
The different expressions of SELENBP1 in head and neck squamous cell carcinoma (HNSCC) detected by IHC. All magnifications 400×. (A) Representative images of immunohistochemistry (IHC) staining for SELENBP1 expression is very low (−) in HNSCC. (B) Representative images of IHC staining for SELENBP1 expression is low (±) in HNSCC. (C) Representative images of IHC staining for SELENBP1 expression is high (+) in HNSCC. (D) Representative images of IHC staining for SELENBP1 expression is very high (++) in HNSCC.

SELENBP1 located at cytoplasmic and nucleus, the intensity and frequency of IHC staining in all tissue sections was analyzed. We integrated intensity and frequency into IHC index for a single classification. Figure 2 shows different expressions of SELENBP1 in HNSCC detected by IHC. Those images of IHC staining for SELENBP1 in HNSCC are diversity, we are divided different expression into two categories: SELENBP1 (−), including low expression, very low expression and SELENBP1 (+), including high expression, very high expression. A comparative analysis revealed that SELENBP1 (−) predominated in HNSCC, and has statistically significant difference comparing with SELENBP1 (+).

### 3.3 SELENBP1 expression in cancer nests is lower than surrounding normal tissue

**Figure 3:**
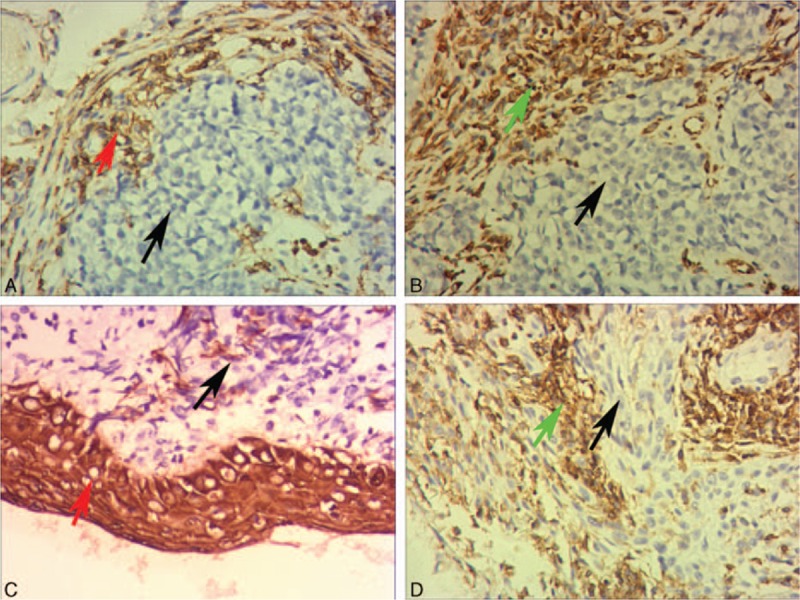
The expressions of SELENBP1 compared to surrounding tissue in HNSCC detected by (IHC). All magnification 400×. Black arrow represents nasopharyngeal carcinoma cells. Red arrow represents normal mucosa cells. Green arrow represents normal lymphocytes. (A,C) Representative images of IHC staining for SELENBP1 in cancer nest comparing to normal mucosa. (B,D) Representative images of IHC staining for SELENBP1 in cancer nest comparing to peripheral tissue.

To determine SELENBP1 expressions in cancer nests comparing with surrounding normal tissue, we chose 3 surrounding normal tissues as control, including normal nasopharyngeal mucosa, normal mucosa on the surface of cancer, and peripheral lymphoid tissue of the nests. The results of IHC indicated that SELENBP1 expression in most of cancer nests is lower than in surrounding normal tissue (Fig. 3).

### 3.4 SELENBP1 expression in different HNSCC has significant differences

**Figure 4:**
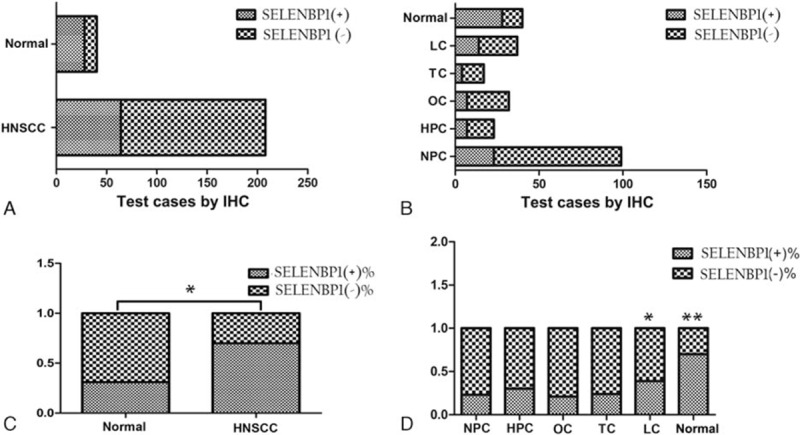
The result of SELENBP1 expressions in HNSCC and normal tissues. (A and B) Results of SELENBP1 expressions in total head and neck squamous cell carcinoma (HNSCC) and normal tissues. (C and D) Results of SELENBP1 expressions in respective HNSCC and normal tissues.

Five kinds of pathological specimens of HNSCC were examined by IHC, including NPC, LC, OC, TC, and HPC. In total, the expression of SBP1 is lower in these tumors comparing with normal tissues. As mentioned in previous literature, different HNSCCs have obvious distinguish in the pathological type, the malignant degree and prognosis, the expression of SELENBP1 also has difference in our research, the expression of SELENBP1 in LC is relatively high, corresponding with the lower malignant and better prognosis (Fig. 4).

### 3.5 SBP1 expression is associated with prognosis of NPC patients

**Figure 5:**
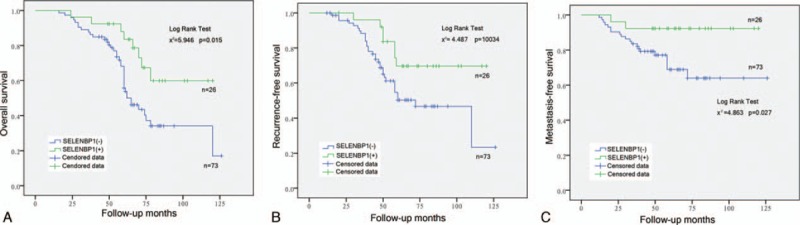
Expression of SELENBP1 in nasopharyngeal carcinoma (NPC) is associated with the prognosis of patients. (A) The low expressions of SELENBP1 is associated with overall survival of patients with NPC. (B) The low expressions of SELENBP1 is associated with tumor recurrence-free survival of patients with NPC. (C) The low expressions of SELENBP1 is associated with tumor metastasis–free survival of patients with NPC.

In our study, 99 NPC patients were classified into 2 subgroups using the IHC SELENBP1 scores, SELENBP1 (+) subgroup (n = 26) and SELENBP1 (−) subgroup (n = 73), then we tried to analyze metastasis-free survival, recurrence-free survival, and OS rate of 2 subgroups. The result indicated that SELENBP1 (−) subgroup has poor OS, tumor recurrence-free survival, and metastasis-free survival of patients with NPC, and has statistically significant (Fig. 5).

### 3.6 SELENBP1 expression has no correlation with tumor N-stage, T-stage, and tumor grade

**Figure 6:**
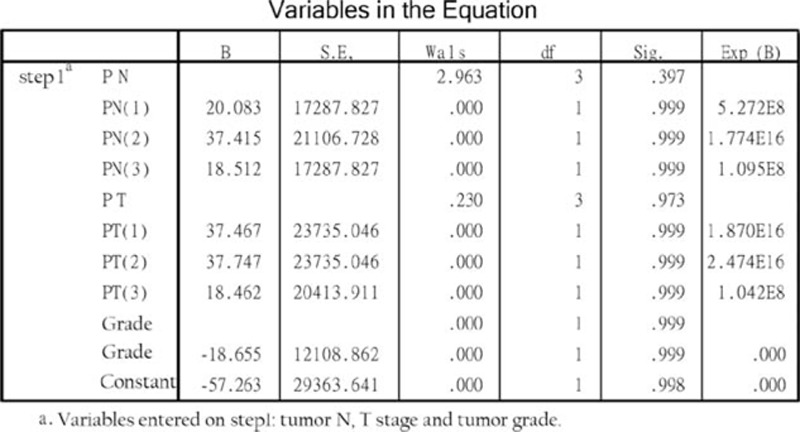
The correlation of the SELENBP1 expression and tumor N-stage, T-stage, and tumor grade.

In previous report of GEO dataset, SELENBP1 expression in HNSCC tissues has no correlation with tumor T-stage, N-stage, and tumor grade, we analysis the relevance that tumor N-stage, T-stage and tumor grade of 99 patients with NPC and SELENBP1 expression with Binary logistic regression analysis, the results were verified from clinical again (Fig. 6).

## 4 Discussion

In our first study, analysis of GEO dataset showed that expression of *SELENBP1* gene in general cancer and HNSCC has significant downregulation different from normal tissues, according to some literature reports.^[22,23]^ SELENBP1 expression in the beginning and advance stage of tumor has obvious difference relative to tumor margin, but between the 2 has no difference. Further analysis shows that SELENBP1 expression has no correlation with T-stage or N-stage or tumor grade. Even though some researchers noted the reduction of SBP1 is likely to occur in the later stages of tumor development in gastric carcinoma^[24]^ and SBP1 levels were shown a trend toward a progressive loss with increasing tumor size in the case of uterine leiomyomas.^[25]^ But we just observed that the change in SELENBP1 expression occurs in the stage of tumorigenesis and no further changes occur with the progress of the cancer. During the process of occurrence and development of tumor, the gene expression is interfered and influenced with each other, so that is complicated and varied in DNA, RNA, and protein expression level, but SELENBP1 expression has not been regulated by other factors, which suggested its expression located in upstream of tumor gene regulation and plays a leading role in the initiation and development of the disease, so it is suggested that SELENBP1 is involved in cancer prevention and treatment of trace element selenium.

SELENBP1 expression of 208 samples by immunohistochemical showed 2 main characteristics, that is relatively lower expression and diversity expression in HNSCC. There are 3 aspects to confirm relatively lower expression: significant low expression in major HNSCC cells significantly and high expression in normal contrast pathological section, as shown in Figure 4; SELENBP1 expression in cancer nests of HNSCC is significantly lower than in surrounding normal epithelium, as shown in Figure 3A and C; SELENBP1 expression in cancer nests of HNSCC is significantly lower than in surrounding normal peripheral lymphocytes, as shown in Figure 3 B and D. Meanwhile, we also found expression of SELENBP1 shows the characteristics of diversity: lower expression in major HNSCC, but still visible some high expression; high expression in normal contrast pathological sections and also visible lower expression in those sections; low expression of SELENBP1 in NPC nests is the mainstream, but we can also see a few medium and high expressions, as shown in Figure 2B–D. So we can draw the following conclusions: the general trend of SELENBP1 expression in HNSCC is downregulated significantly, but lack of specificity and sensitivity, so SELENBP1 expression level has relatively limited value in the diagnosis of HNSCC, but may be more significant to assess patient prognosis.

In our study, 99 patients of carcinoma were followed up in detail and these paraffin specimens were analyzed by IHC: SELENBP1 has negative expression in most of NPCs and the patients with negative expression have poor OS compared with positive expression (Fig. 5A). To clarify the relationship between SELENBP1 and OS in tumor patients, several retrospective studies showed that SBP1 expression in tumor cells was an independent risk factor for both OS and recurrence in patients with gastric carcinoma,^[9]^ lung adenocarcinomas,^[11]^ colorectal carcinomas,^[12]^ hepatocellular carcinoma,^[7]^ and breast carcinoma.^[26]^ Some authors conducted in-depth research in culture cell and animal, decrease of SELENBP1 expression promoted cell proliferation and migration, suppressed cell differentiation correlate with vascular invasion in vitro.^[7]^ In contrast, increasing SBP1 expression could lead to senescence, inhibition of cell proliferation, migration, and tumor growth,^[8,27]^ and these results also have been validated in animals. We further analyzed the relationship between SELENBP1 expression and recurrence or metastasis of NPC; the results show that low SELENBP1 expression was associated with high tumor recurrence and metastasis (Fig. 5B and C), indicating that negative SBP1 expression could be a potential biomarker predicting early recurrence or poor prognosis in NPC patients, so the internal mechanism is worth further research and exploration. We also analyzed the relationship between SELENBP1 expression and tumor N-stage, T-stage, and tumor grade, as a result has nothing to do (Fig. 6), so indicating that SELENBP1 expression does not reflect the degree of cancer development, and it is consistent with analysis of GEO data. In a word, SELENBP1 expression is a related factor for speculating the prognosis of NPC patients, rather than the progress.

## 5 Conclusion

Overall, our study reveals that SELENBP1 expression in HNSCC comparing to normal tissue has significant difference. SELENBP1 expression is closely related to the prognosis of NPC patients, but also may be a more effective predictor for tumor recurrence or metastasis, and such a conclusion guides our follow-up treatment. Therefore, reduced SELENBP1 may play a critical role in regulating malignant transformation and cancer progression; further investigations of model organisms to define the biological functions of SELENBP1 and the effects on tumor cells are required to further elucidate these complexities.
